# RaMP: A Comprehensive Relational Database of Metabolomics Pathways for Pathway Enrichment Analysis of Genes and Metabolites

**DOI:** 10.3390/metabo8010016

**Published:** 2018-02-22

**Authors:** Bofei Zhang, Senyang Hu, Elizabeth Baskin, Andrew Patt, Jalal K. Siddiqui, Ewy A. Mathé

**Affiliations:** 1 Department of Biomedical Informatics, College of Medicine, The Ohio State University, Columbus, OH 43210, USA; zhang.5675@buckeyemail.osu.edu (B.Z.); hu.993@buckeyemail.osu.edu (S.H.); baskin.18@buckeyemail.osu.edu (E.B.); patt.14@buckeyemail.osu.edu (A.P.); Jalal.Siddiqui@osumc.edu (J.K.S.); 2 Biomedical Engineering Undergraduate Program, The Ohio State University, Columbus, OH 43210, USA; 3 Biomedical Sciences Graduate Program, The Ohio State University, Columbus, OH 43210, USA

**Keywords:** pathway analysis, metabolomics, transcriptomics, pathway database

## Abstract

The value of metabolomics in translational research is undeniable, and metabolomics data are increasingly generated in large cohorts. The functional interpretation of disease-associated metabolites though is difficult, and the biological mechanisms that underlie cell type or disease-specific metabolomics profiles are oftentimes unknown. To help fully exploit metabolomics data and to aid in its interpretation, analysis of metabolomics data with other complementary omics data, including transcriptomics, is helpful. To facilitate such analyses at a pathway level, we have developed RaMP (Relational database of Metabolomics Pathways), which combines biological pathways from the Kyoto Encyclopedia of Genes and Genomes (KEGG), Reactome, WikiPathways, and the Human Metabolome DataBase (HMDB). To the best of our knowledge, an off-the-shelf, public database that maps genes and metabolites to biochemical/disease pathways and can readily be integrated into other existing software is currently lacking. For consistent and comprehensive analysis, RaMP enables batch and complex queries (e.g., list all metabolites involved in glycolysis and lung cancer), can readily be integrated into pathway analysis tools, and supports pathway overrepresentation analysis given a list of genes and/or metabolites of interest. For usability, we have developed a RaMP R package (https://github.com/Mathelab/RaMP-DB), including a user-friendly RShiny web application, that supports basic simple and batch queries, pathway overrepresentation analysis given a list of genes or metabolites of interest, and network visualization of gene-metabolite relationships. The package also includes the raw database file (mysql dump), thereby providing a stand-alone downloadable framework for public use and integration with other tools. In addition, the Python code needed to recreate the database on another system is also publicly available (https://github.com/Mathelab/RaMP-BackEnd). Updates for databases in RaMP will be checked multiple times a year and RaMP will be updated accordingly.

## 1. Introduction

Metabolomics is undeniably powerful for uncovering disease biomarkers [1,2,3]. Beyond biomarker discovery though, metabolomics data can provide information on biological mechanisms that are disrupted in diseases. From an analysis point of view, identifying these biological roles is very challenging, and typically requires integration of additional molecular information, such as other omics and biological pathway annotations [4]. Nonetheless, analysis of metabolomics data with other omics data, such as transcriptomics, has uncovered relevant gene-metabolite associations and disease-relevant metabolic functions and pathways [5,6,7,8,9]. Finding genes associated with metabolite levels, or whose products catalyze reactions involving disease-related metabolites, or their associated pathways, can generate hypotheses on how these metabolic phenotypes are regulated. In turn, these hypotheses could elucidate functional mechanisms that could be targeted to generate a desired metabolomics phenotype. Understanding the regulation of metabolic phenotypes will expand knowledge of disease biology, and could contribute to finding successful interventions, including accurate predictions of diagnosis, prognosis, and treatment outcomes.

While numerous methods and approaches that integrate gene expression and metabolomics data have been reported [10,11,12], public and web-accessible software packages that integrate these data are generally sparse. Furthermore, these tools are often tailored to specific analysis types, such as pathway visualization, pathway enrichment and overrepresentation analysis, network analysis or reaction-level/metabolic flux analysis. Of these, MetaboAnalyst 3.0 [13], IMPaLA [14], XCMS [15,16], and Metabox [17] integrate metabolomics and gene expression for pathway enrichment and/or network analysis (Table 1). In addition, Pathway Commons [18] integrates many sources of pathway annotations and includes functionalities for pathway analysis on genes (Table 1). While Metabox, XCMS, and MetaboAnalyst primarily use KEGG annotations, the other tools combine multiple databases. Combining multiple databases is advantageous as it broadens the scope of genes and metabolites that have pathway annotations. However, these combined databases are not readily accessible, making it difficult, if not impossible, to query and to integrate with improved analysis tools. Furthermore, statistics used in these software assume that pathways are independent of each other. This assumption is false since the hierarchical nature of many databases (e.g., KEGG, Reactome) yield pathways that overlap each other in terms of the genes and metabolites contained therein. Also, there are content overlaps between pathways that are drawn from various database sources. 

To address these limitations, we developed RaMP (Relational database of Metabolomics Pathways), a publicly available, comprehensive database of gene and metabolite pathways. RaMP is carefully designed to enable complex searches across genes and metabolites (e.g., find genes involved in regulating key metabolites), and across distinct types of annotations, such as biofluid location, disease, and biological pathways (e.g., find metabolites detected in urine and involved in cancer). This design also allows analysis of pathway content overlap for development of improved pathway enrichment statistics. RaMP is publicly available at https://github.com/mathelab/RaMP-DB/ and can be used in two different ways: (1) it can be downloaded as a mysql dump (https://github.com/mathelab/RaMP-DB/inst/extdata/), for integration into any other tool; (2) it can be accessed via a user-friendly R Shiny web interface that supports basic queries, enrichment analysis given a list of genes and metabolites, and network visualization of gene-metabolite relationships. Overall, RaMP provides an up-to-date, comprehensive gene and metabolite pathway annotations that can be used as a stand-alone resource or can readily be incorporated into other tools. It is our hope that this resource will improve biological interpretation of metabolomics phenotypes, will guide data-driven hypothesis generation on the modulation of these phenotypes, and will thus advance scientific knowledge of metabolic phenotypes.

## 2. Results

### 2.1. RaMP Design

A multi-database integration approach has been successfully applied for gene/metabolite enrichment analysis [14,19,20,21], yet their underlying databases are not downloadable, do not allow complex or batch queries, or do not account for pathway redundancy in their statistical enrichment metrics. To facilitate development of improved pathway analysis methods and tools, RaMP is publicly available and incorporates the following publicly available databases: KEGG [22,23,24], Reactome [25,26], HMDB [27,28,29], and WikiPathways [30,31,32]. The KEGG database was chosen because it is one of the most widely used and complete pathway databases. The KEGG “Human maps”, that represent manually curated human diseases and molecular interactions from various organisms (experimental evidence in specific organisms are generalized to others), are incorporated into RaMP. HMDB is the largest collection of annotations for small molecules found in humans, and is thus the more complete resource for metabolite annotations. HMDB provides links to SMPDB [33,34] and KEGG pathway databases. Only the SMPDB pathways from HMDB are incorporated into RaMP, since KEGG pathways are integrated directly through the KEGG REST API. HMDB information about diseases, biospecimen location, and synonyms is also input into RaMP. We further included information about genes and metabolite pairs that are involved in the same reaction (e.g., “enzymes” section in HMDB entries). 

Reactome pathways were included because they are derived from published experimental evidence and are curated by expert molecular biologists. Reactome also contains relevant disease pathways. The hierarchy in Reactome is such that the lowest level pathways represent single reactions, which is important for retrieving the gene(s) that catalyze reactions involving metabolites of interest. Finally, we incorporated WikiPathways because it is one of the largest human pathway collections to date and has recently undergone considerable growth in metabolic pathway annotations [31,32]. Importantly, WikiPathways updates its content, both through individual users and groups from the general scientific community through the Wiki. WikiPathways are curated for quality and only those pathways that pass the curators’ quality metric are included into RaMP. 

**Figure 1:**
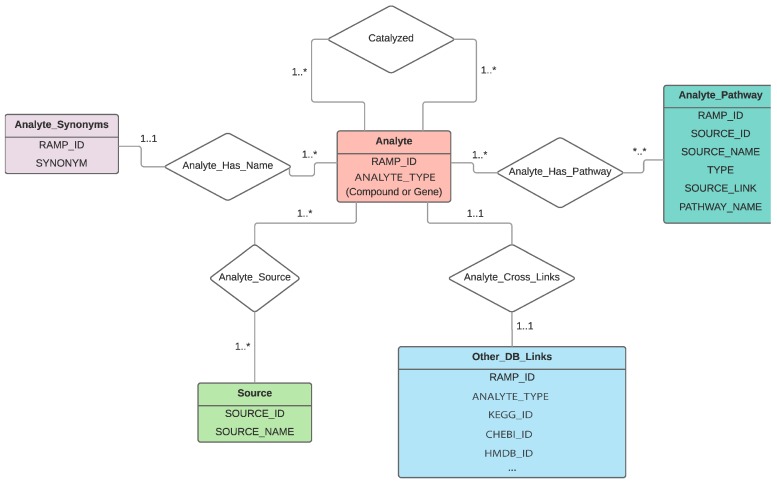
Schema of the database, depicting the tables included in the database and how they are related.

Because the intent of RaMP is to retrieve biological pathways that relate genes and metabolites, the logical relationship between genes, metabolites, and associated pathways can be identified upfront and naturally yields a relational structure. RaMP is thus written in MySQL. The Python code used to pull in the data from each individual database is publicly available at https://github.com/Mathelab/RaMP-BackEnd. Importantly, the design of the database (Figure 1) is centered on the analytes (genes or metabolites), not on the pathways. The main reason for this design is to readily retrieve genes and metabolites that belong to the same pathway or reactions. This design also facilitates complex queries across multiple annotations (genes, metabolites, pathways). Equally important, an internal RAMP ID is attributed to each gene, metabolite, and pathway (see Methods). One issue with metabolite and gene names is that there are many synonyms for individual analyte names. Creating unique IDs based on synonyms is not possible, because there are synonym names that are commonly used for many different metabolites and genes. For example, the synonym “triglyceride” is used for all the triglycerides in HMDB, of which there are 13,919. When populating the RaMP database, a unique RaMP ID is attributed to database compound IDs that are linked to each other. To help ensure that RaMP IDs map to a unique metabolite (e.g., there are no multiple RaMP IDs for the same metabolite), we check whether a database compound ID is already attributed to a RaMP ID for every new database compound ID that is processed. For example, glucose has one unique RaMP ID, but is found in multiple databases and is thus linked to multiple database IDs: ChEBI ID 4167, PubChem Compound ID 3333, KEGG ID C00031, and HMDB ID HMDB0000122. A similar procedure is applied for internal pathway RaMP IDs. The list of IDs and other information (e.g., synonyms) retrieved from each database is listed in Table S1. See Methods for information regarding the mapping of IDs from different databases.

### 2.2. RaMP Content

The number of genes, metabolites, and pathways in each database are shown in Table 2. In total, RaMP integrates 51,526 pathways (from KEGG, Reactome, SMPDB, and WikiPathways), 23,077 genes, and 113,725 metabolites. Furthermore, 157 ontologies from HMDB have been incorporated, including biofluid type (e.g., blood, urine, etc.), cellular location (e.g., nucleus, mitochondria, etc.), origins (e.g., drug, food, microbial, etc.), and tissue location (e.g., teeth, lung, etc.). Gene and metabolite pairs that are involved in the same reactions are retrieved from the HMDB database. 

**Figure 2:**
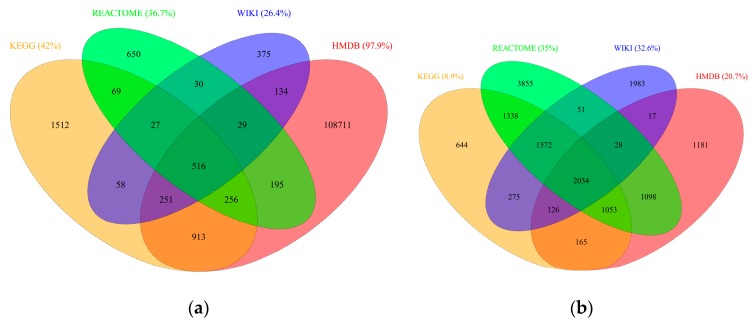
Overlap of (**a**) metabolites and (**b**) genes within each database integrated into RaMP.

Importantly, integration of the four databases into RaMP widens the coverage and variety of metabolites and genes that have pathway annotations. Figure 2a,b depict the number of overlapping metabolites and genes, respectively, among the four databases integrated into RaMP. Only a small fraction, 0.05% of metabolites and 13.2% of genes, overlap between all four databases. This relatively low overlap is not surprising given the fact that the four databases were constructed using varying input resources and for different purposes, as described above. Nonetheless, the low overlap exemplifies the strength in integrating annotation databases to increase the number of metabolites and genes of interest that map to pathways. In fact, each database has a high percentage of analytes that are unique to that database: 42% metabolites and 8.9% genes in KEGG, 36.7% metabolites and 35% genes in Reactome, 26.4% metabolites and 32.6% genes in WikiPathways, and 97.9% and 20.7% genes in HMDB. It is important to note that HMDB contains many metabolites that do not map to pathways (of the 111,105 metabolites incorporated into our RaMP database, 48,623 or 43.8%, are mapped to a KEGG or SMPDB pathway).

When assessing the number of pathways each metabolite is involved in, a few hundred metabolites are involved in many pathways (Figure S1). For example, 5′ (Tetrahydrogentriphosphate) Adenosine, Adenosindiphosphorsaeure, and dihydrogenoxide are involved in over 600 pathways in the Reactome database. This promiscuity may render interpretation of pathway analysis more complicated because many more hits could be returned if a promiscuous metabolite is involved, yet it is unlikely that all these pathways are involved simultaneously. Flagging these metabolites when performing pathway enrichment analysis could be beneficial, unless the specific context of the system under study is well defined (e.g., specific cells, cellular localization, disease, etc.).

### 2.3. Pathway Redundancy and Clustering of Enriched Pathways

**Figure 3:**
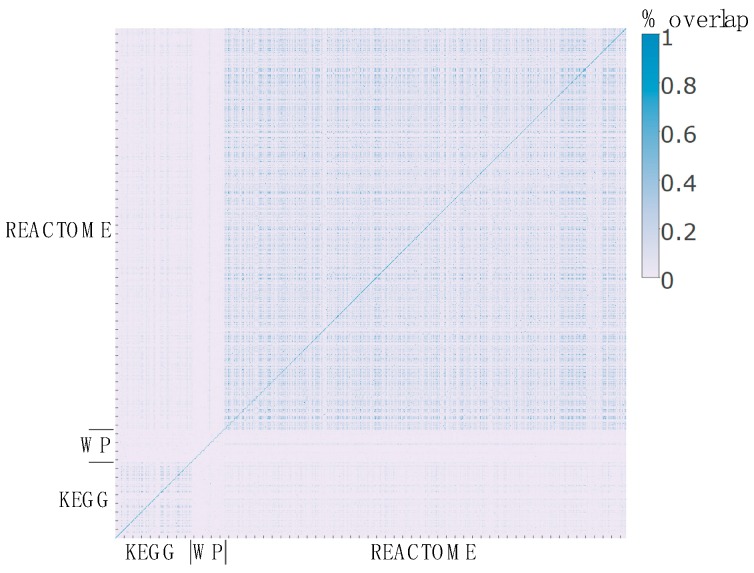
Percentage of metabolite overlap in each pathway from all databases that are integrated in RaMP. (WP–WikiPathways).

Integration of databases enables redundancy analysis, where the goal is to evaluate how much overlap in genes or metabolites exists between pathways that are present in different databases. Figure 3 depicts the metabolite percent overlap (Number of metabolites in common/union of all metabolites in two pathways being compared, see Methods) for all pairwise comparisons of pathways from KEGG, Reactome, and WikiPathways incorporated into RaMP. Pathways within Reactome and KEGG show the largest number of overlapping pathways. For Reactome, these overlaps are likely to reflect the hierarchical structure of pathways. As an example, the “Formation of COPII vesicle” pathway in Reactome is a subpathway of “MHC class II antigen presentation”, which is a subpathway of the “Adaptive Immune System” pathway. In contrast, the overlap in gene content between pathways is much less compared to that of the overlap in metabolite content (data not shown). 

**Figure 4:**
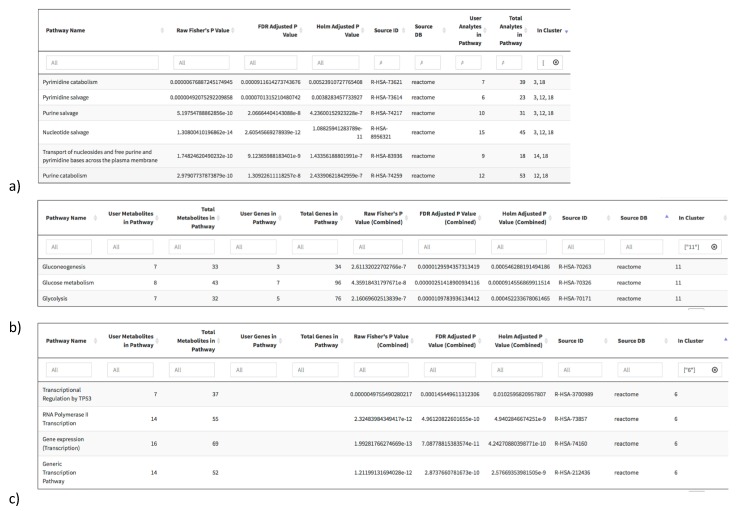
Output from pathway overrepresentation analysis using the RaMP R package web application. Significant pathways are derived from a list of metabolites and genes that are altered in breast tumor tissue relative to adjacent tumor tissue in a publicly available breast cancer dataset (see Methods). (**a**) Nucleic acid metabolism cluster of statistically significant pathways resulting from analysis using metabolites as input. (**b**) Glucose metabolism and (**c**) transcriptional regulation pathway clusters resulting from analysis using metabolites and genes as input.

Content overlaps of pathways within or between databases can make interpretation of pathway enrichment analyses confusing. To address this, we have implemented a clustering approach, based on a heuristic fuzzy multiple-linkage partitioning algorithm [35], to group findings by functional homology (see Methods for further details). To demonstrate this utility, we have analyzed a list of altered metabolites and genes between breast tumor tissue and adjacent non-tumor tissue from a previously published study [5] (see Methods, Figure 4). When performing pathway overrepresentation analysis, the RaMP package outputs enriched pathways that can be sorted by p-value or database source (e.g., all significant pathways from KEGG are grouped, then pathways from Reactome, etc.). Next, we clustered these pathways and identified high levels of overlap between significant pathways. This clustering thus allows the user to quickly sort through redundant results and identify functionally relevant pathways. In the altered breast cancer metabolite data set, our clustering algorithm identified a relevant cluster of pathways involved in nucleic acid metabolism (Figure 4a). It is well documented that various cancer types induce shifts in de novo nucleotide synthesis, catabolism, and nucleoside salvage [36]. When both genes and metabolites were input into our algorithm, clusters of glucose metabolism and transcriptional pathways were significant (Figure 4b,c). These enriched clusters are concordant with previous work reporting that cancer cells undergo higher rates of aerobic glycolysis (“Warburg effect”) [37] and alterations of the transcriptional machinery with TP53 being among the most mutated in cancers [38]. As the pathways identified in one cluster contain >50% overlap in their metabolite/gene composition, it is clear that enrichment of these pathways is driven by their common metabolites. This pathway clustering thus offers a flexible way to improve interpretability of results by identifying groups of pathways with many genes and metabolites in common, allowing users to quickly and efficiently identify functional groups of interest. 

### 2.4. RaMP Access and User Interface

Access to the code used to build the RaMP MySQL database, the RaMP database itself (mysql dump), and the associated R package are publicly accessible on our GitHub site https://github.com/mathelab/RaMP-DB. Instructions for creating the MySQL database locally and running the R package are detailed on the front page of the GitHub site. For users that want to perform basic queries and pathway enrichment analysis without programming overhead, we have developed an R package that includes an R Shiny web interface (see Supplementary Material for installation instructions). The package can be readily installed using the devtools R package with the command install_github(“mathelab/RaMP-DB”). 

Once installed, the application runs by simply typing “runRaMPapp (password = ”mysqlpassword”)” in the R console. The interface supports 4 basic types of queries (Table 3) that can be run in batch: (1) Given a list of pathway(s), retrieve all analytes involved; (2) Given a list of analyte(s), retrieve the pathways that each analyte(s) is involved in; (3) Given a list of analytes, return the analytes that are involved at a reaction level (e.g., return metabolites catalyzed by user-input genes, based on HMDB database); (4) Given a list of ontologies or metabolites, retrieve the corresponding metabolites or ontologies, respectively. In addition to queries, the web application supports pathway overrepresentation analysis on genes, metabolites, or genes and metabolites combined, and results can be grouped by database type or clustered by pathway overlap, as described above. This pathway analysis is embedded in the second query (retrieve pathways from a user-input list of analytes). Furthermore, the web application provides network visualization of gene-metabolite relationships that are retrieved from a user-input list of genes or metabolites (query 3, Figure S2). The Supplementary Materials provides details on how to utilize the web app, and includes snapshots of each query.

## 3. Discussion

One of the first steps in statistical analysis of metabolomics data is to identify metabolites that are altered between disease states or conditions under study. This step however is oftentimes insufficient to fully leverage the data and understand the underlying biological mechanisms at play. To provide such further insights, one can combine metabolomics data with other data, such as gene expression and pathway annotations. To facilitate such integration at a pathway level, we have developed the relational database RaMP, which incorporates gene and metabolite pathway annotations from four large, and commonly leveraged databases: HMDB, KEGG, Reactome, and WikiPathways. RaMP was designed to allow complex and batch queries, to facilitate integration with other tools, and to provide improved pathway overrepresentation functionality. The relational structure supports complex and batch queries, and the publicly available MySQL dump (https://github.com/mathelab/RaMP-DB/inst/extdata/) enables advanced users to easily set up the database locally. We have improved interpretation of pathway enrichment analysis by calculating pathway overrepresentation using 3 databases (KEGG, Reactome, WikiPathways) in RaMP, and by providing different groupings of enriched pathways (by database origin or pathway overlap). Furthermore, all the underlying Python code used to create the RaMP MySQL file is publicly available (https://github.com/Mathelab/RaMP-BackEnd), thereby ensuring full transparency of the database construction, and complying to reproducibility best practices. Lastly, we have wrapped RaMP into an R package that contains a user-friendly web interface for performing several queries and pathway overrepresentation analysis. The R package is publicly available on GitHub at https://github.com/mathelab/RaMP-DB/, where detailed installation instructions are provided. 

As with any research endeavor, RaMP has limitations. One current issue is the integrity of mapping metabolite names to an appropriate compound ID. Mapping can be hampered because there are synonyms that are generalized compound names and thus map to a large number of metabolites. One extreme example is “triglyceride”, which maps to 13,719 different compound IDs. Further, there are synonyms that have different IDs even though they correspond to different levels of structure resolution, which is highly dependent on the platform. For example, some platforms can distinguish isomeric structures (2,3-Dimethylphenol vs 2,5-Dimethylphenol) while others cannot. One existing solution to this problem is the Metabolomics Workbench Refmet resource [19] that provides a translation service that retrieves a common, “lowest denominator” name for each compound, thereby facilitating harmonization of names across platforms. This type of harmonization could be integrated into RaMP for improved metabolite mapping when the metabolites under study are present in Refmet. Ultimately though, it is important for the users to check that the mapping of IDs is correct.

In addition, the background number of metabolites used to calculate pathway enrichment is based on the number of metabolites represented in each pathway database (e.g., 4134 metabolites mappable to KEGG pathways). The default number of genes used for background is set to 20,000. In the future, users will have the option to provide a list of genes or metabolites assayed to build a custom contingency table for the test. This capability is particularly relevant for analysis of metabolites, where the number of metabolites measured in a given experiment is variable. Because RaMP is continuously being developed, we anticipate expansion of the RaMP functionalities to increase utility and usability. In addition to the aforementioned pathway enrichment changes, we also plan to develop more query capabilities. Furthermore, while overrepresentation analysis can be useful for uncovering disrupted biological pathways, we recognize the existence of improved, second and third generation methods that take into account topology [39,40,41], and pathway dependency and crosstalk [42]. With the accessibility and organization of RaMP, it is our hope that incorporation of up-to-date and comprehensive annotation of genes and metabolites into improved pathway analysis methods will be facilitated. Future developments of RaMP will include expansion of RaMP pathway analysis approaches and functionalities to increase utility and usability. 

While RaMP is currently focused on human pathways, we plan to expand the database to other organisms. In particular, with the increasing appreciation of the impact of microbial metabolites on human metabolism, microbial pathway databases could be integrated into RaMP to further expand its utility for integrative pathway analysis. With this in mind, it is important to note that the content of RaMP revolves around analytes (genes and metabolites) and how they are related (pathway involvement, reaction-level relationships). Therefore, when information from source databases (HMDB, KEGG, Reactome, WikiPathways) is included, only information that pertains to downstream pathway enrichment analysis is retained. With this mindset, we hope to retain the simplicity of our database design (Figure 1). 

In conclusion, RaMP is a standalone database and application, usable through a web interface that was developed to facilitate gene and metabolite pathway analysis. RaMP can be used independently as a MySQL database that can be readily integrated with other tools, or can be accessed through our R package and web interface. RaMP is thus a first step toward a comprehensive integration of genes and metabolites at a pathway level, and it is our hope that our transparent approach, with all code publicly available, will generate further developments and improvements toward more complete interpretation of metabolomics data.

## 4. Materials and Methods

### 4.1. Parsing Raw Database Files

All metabolite and pathway data were downloaded from HMDB, KEGG, Reactome, and WikiPathways using Python scripts, including Python library urllib, based on HTTP protocol. All the code is available at https://github.com/Mathelab/RaMP-BackEnd. Because the format of the data varies by database, individual classes and parsing procedures were created for each database

The HMDB data, in Extensible Markup Language (XML) format, was parsed using the Python built-in parser from the ElementTree XML API. First, the HMDB ID is retrieved through the “metabolite” tag of the XML file. Next, for each “metabolite” tag, information for other tags are retrieved, including gene names and IDs, pathway names, and other ontologies (biofluid location, cellular location, origin, and tissue location). While parsing, dictionaries are created where the keys are HMDB IDs and the associated values are all available attributes (e.g., synonyms, genes involved in metabolite reactions, pathways, etc.) pertaining to that metabolite. 

The KEGG data was retrieved through the REST API as “txt” files, and each file type was parsed in the following order: pathways, metabolites, metabolite synonyms, genes, and gene synonyms. To use the REST API, the complete list of human pathway IDs (http://rest.kegg.jp/list/pathway/hsa) was used to retrieve information on the pathways and associated genes and metabolites. For example, information on the first pathway in the complete list of human pathway IDs, “hsa00010”, is accessible through the link http://rest.kegg.jp/get/hsa00010. Parsing compound and gene IDs from this pathway entry allows us to retrieve further information on the compounds and genes related to that pathway (e.g., metabolite http://rest.kegg.jp/get/C00022 and gene http://rest.kegg.jp/get/hsa:3101).

For WikiPathways, the data are stored in a GenMAPP Pathway Markup Language (GPML) format, which is a custom XML format compatible with pathway analysis tools such as Cytoscape, GeneMAPP and PathVisio. This file format retains all of characteristic of XML, so we apply the same procedure used for parsing the HMDB database.

Finally, the physical entity identifier mapping files that map compound (ChEBI) IDs and gene (UniProt) IDs to Reactome pathways were downloaded from Reactome. Each file is tab-delimited and 3 columns are retrieved: (1) compound/gene identifiers; (2) Reactome pathway ID; (3) Reactome pathway name; (4) genes and species. As with the other databases, only human pathways were selected. The Python library “libChEBI” is used to retrieve the ChEBI common name from each ChEBI ID retrieved from Reactome. Similarly, the gene common names are retrieved through the UniProt REST API. 

### 4.2. Creating Unique RaMP IDs

Metabolite and gene names have many synonyms and sometimes, the synonyms can be the same for different molecules. Furthermore, different databases use different identifiers. To properly map identifiers from one database to the next, we (1) created dictionaries of IDs for each database source and (2) ensured that identifiers linked to common IDs had the same RaMP ID. In the first step, source IDs were used as the key in the dictionaries and the values were the other identifiers present in the source database (see Supplementary Table S1). In the second step, the dictionaries are parsed and a RaMP ID is created for each new ID that is encountered. A two-column table that relates RaMP IDs with source IDs (one RaMP ID to many source IDs) is created. For each new key (source ID) in the dictionaries, the associated values and the value of the key itself are searched against the RaMP ID/source ID table. If there is a match, then all values for that key (including the key itself) are assigned to the matching RaMP ID. If there is no match, then a new RaMP ID is created and all values are assigned to the new RaMP ID. An analogous approach is used for pathways and ontologies. Of note, it is possible that ID mappings from different databases for the same metabolite or gene do not have any overlap. For such cases, these ID mappings would have different RAMP IDs.

RaMP IDs have a prefix, followed by a unique number. The prefix “RAMP_C” is used for compounds, “RAMP_G” for genes, “RAMP_P” for pathways, and “RAMP_OL” for ontologies. Prefixes are then concatenated to a number (from “000000001” to “999999999”). While RaMP IDs are created to map metabolites and genes appropriately across the different databases, these IDs are internal and are not returned to the user through the R package. 

### 4.3. R Package

The R package for RaMP is available online via GitHub (https://github.com/mathelab/RaMP-DB/). Instructions are provided on how to set up MySQL and the RaMP database on this GitHub site. The RaMP R package can be installed via the install_github() command from the devtools package and requires R (≥3.2.0). Questions and concerns can be raised as issues on the GitHub site. Further documentation is provided in the Supplementary Material on how to run the application. 

### 4.4. Pathway Overrepresentation Analysis

RaMP supports pathway overrepresentation analysis of user-supplied lists of metabolites and/or genes. Fisher’s exact tests are performed to calculate pathway overrepresentation *p*-values for metabolites (P_m_) and genes (P_m_), independently. Of note, if pathways contain only genes or only metabolites, then P_m_ or P_g_, respectively, cannot be computed. A combined *p*-value (P_comb_) is then calculated for pathways that are annotated with both genes and metabolites, using Fisher’s method [43]. Specifically, *p*-values are combined using Fisher’s combined probability test, where the test statistic, T_comb_ is calculated as:

(1) $$T_{\mathrm{comb}}=-2\times \ln\left(P_{m}\right)+\ln(P_{g})\;$$

T_comb_ follows a χ^2^ distribution with 2 degrees of freedom and the associated p-value, P_comb_, is calculated using the R function pchisq() and 2 degrees of freedom. When P_m_ is missing, P_comb_ = P_g_. Conversely, when P_g_ is missing, P_comb_ = P_m_. Resulting P_comb_ *p*-values are adjusted for multiple comparisons using the Benjamini and Hochberg method and the Holm method to control the false discovery rate. Similar to other approaches [13], the default total number of metabolites to be used as background is set to the number of metabolites mappable to pathways in each database (3603 for KEGG, 1771 for Reactome, and 1421 for WikiPathways). In the future, we will support a user-input list of metabolites to be used as background. For genes, the total number of genes used as background is 20,000. Pathways derived from KEGG, Reactome, and WikiPathways are used for pathway enrichment analysis, and pathways with <10 or >1000 analytes are removed since those are either too narrow or too broad for meaningful interpretation.

### 4.5. Clustering of Pathway Enrichment Analysis Results

By default, pathway enrichment analysis results are returned for each database (KEGG, Reactome, WikiPathways), ordered by the database the enriched pathway was found in. To improve interpretability of pathway analysis results, enriched pathways are placed in groups according to the proportion of analytes they share in common, allowing the user to more efficiently navigate through redundant pathways. To accomplish this, we implemented an agglomerative clustering algorithm based on the heuristic fuzzy multiple-linkage partitioning algorithm, which is used by the DAVID gene functional annotation tool [35]. The algorithm is comprised of the following four basic steps:

1. *Calculating analyte overlap*: The degree of analyte overlap was calculated for all possible pairs of pathways. Gene overlap and metabolite overlaps were calculated separately. Given two pathways, *m* and *n*, the overlap score O_mn_ represents the Jaccard index, which is calculated as:
  (2) $$O_{\mathrm{mn}}=\frac{I_{\mathrm{mn}}}{L_{m}+L_{n}-{\;I}_{\mathrm{mn}}}$$
  where I_mn_ is the number of analytes (genes or metabolites) present in both pathways, and L_m_ and L_n_ are the number of total analytes in pathways m and n, respectively. When no analytes are in common between two pathways, O_mn_ = 0. Conversely, O_mn_ = 1 if all analytes overlap between two pathways.
2. *Identifying seeds*: The overlap scores O_mn_ are used to identify cluster seeds. Pathways with a high degree of overlap with multiple other pathways (e.g., ≥30% overlap with at least 2 other pathways) are considered “seeds”. Thresholds for percent overlap and number of pathways to overlap with can be defined by the user.
3. *Initial pathway clustering*: Once seeds are identified, pathways are clustered to the seeds based on the overlap scores. Pathways that have overlap scores with seed pathways greater than or equal to a user-defined threshold (e.g., 30%) are clustered with the corresponding seed pathway. Of note, this approach allows for a single pathway to belong to multiple clusters, as long as it is sufficiently similar to the seed pathway of those clusters.
4. *Calculate cluster overlap*: Overlap scores between clusters are calculated with the same formula as Equation (2), with the following definitions for I and L: I_mn_ is now the number of pathways in common (based on their names) between clusters m and n, and L_m_ and L_n_ are now the number of pathways in clusters m and n, respectively. All pairwise cluster similarities (e.g., cluster overlap scores) are ranked, and the cluster pair with the highest overlap score is merged into a single cluster, provided that their overlap score is greater than a user-defined merge threshold (e.g., 30%).
5. *Repeat cluster overlaps:* Step 4 is repeated until there are no cluster overlap scores above the merge threshold.

With this clustering approach, large and complex lists of enriched pathways are grouped into clusters of highly similar pathways. This feature is important as it allows users to more easily interpret functional implications of pathway enrichment results.

### 4.6. Pathway Analysis in Breast Cancer Dataset

Metabolite data was obtained for a previously published breast cancer study comparing tumor and adjacent non-tumor breast tissue [5]. Metabolites with more than 80% imputed values were filtered out. A t-test was performed on tumor and non-tumor samples and the resulting *p*-values were adjusted using the False Discovery Rate (FDR) method. Metabolites, mappable to KEGG or HMDB IDs, that had a fold-change greater than +/− 1.5 with an FDR adjusted *p*-value <0.05 were then input into the RaMP web application using the “Return pathway from given analytes” tab and the “Input Multiple Metabolites (batch query)” subtab. Overrepresentation analysis was performed on the list of metabolites and pathways were retained if their Holm-adjusted *p*-values were <0.01. Clustering of these pathways was performed using the following parameters: overlap threshold for medoid establishment = 0.2, number of similar neighbors = 2, overlap threshold for cluster merge = 0.75. Overrepresentation analysis was repeated with a list of metabolites and genes as input (Holm-adjusted *p*-values <0.01). Parameters for clustering these pathways were: overlap threshold for medoid establishment = 0.2, number of similar neighbors = 2, overlap threshold for cluster merge = 0.5.

## Figures and Tables

**Table 1:** Tools that support over-representation and pathway enrichment analysis of genes and/or metabolites. These tools include a user-friendly web interface. ORA–Overrepresentation analysis.

Tools	Databases Included	Access and Availability	Batch Queries	Pathway Analysis	Network Visualization/Analysis	Pathway Clustering	Output
RaMP https://github.com/Mathelab/RaMP-DB/	KEGG, Reactome, WikiPathways, HMDB/SMPDB	- R package - MySQL Dump - Python code to build MySQL Dump	Yes	ORA	Yes	Yes	- Interactive tables of query results - Interactive tables of pathway analysis results - Clustering of enriched pathways by pathway similarity
IMPaLA https://impala.molgen.mpg.de	KEGG, Reactome, BioCyC, PID, BioCarta, NetPath, INOH, EHMN, PharmGKB, WikiPathways, SMPDB	Web services programming interface	No	- ORA - Wilcoxon enrichment analysis	Yes	No	- Interactive tables of pathway analysis results with clickable links
MetaboAnalyst https://www.metaboanalyst.ca	KEGG, HMDB, SMPDB	R package	No	- ORA - Metabolite set enrichment analysis - Integrated topology and enrichment analysis (metabolites only) - Integrated gene and metabolite pathway analysis	Yes	No	- Interactive tables and plots of pathway analysis results with clickable links - Interactive pathway viewer
Metabox https://kwanjeeraw.github.io/metabox	KEGG, PubChem, UniProt, ENSEMBL, miRTarBase, BioGRID, Pathway Commons	R package	No	- ORA - Set enrichment analysis	Yes	No	- Interactive tables of pathway analysis results - Interactive visualization of networks (with table of nodes/edges) with clickable links
XCMS https://xcmsonline.scripps.edu/	METLIN, KEGG, HMDB, Lipid Maps, NIST, MassBank	- R package - Web interface	No	Predictive Pathway Analysis	No	No	- Interactive tables of pathway results with clickable links - Interactive pathway cloud plot for visualization
Pathway Commons http://www.pathwaycommons.org	Reactome, NCI PID, PhosphoSitePlus, HumanCyc, HPRD, PANTHER, DIP, BioGRID, intAct, BIND, CORUM, MSigDB, miRTarBase, DrugBank, Recon X, CTG, KEGG, SMPD, INOH, NetPath, WikiPathways, ChEBI, SwissProt, UniChem	- R package - Web services programming interface	No	Gene set enrichment analysis	Yes	No	- Interactive pathway visualization

**Table 2:** Databases incorporated into RaMP, including the number of metabolites, genes, and pathways.

Database	# Metabolites	# Genes	# Pathways	Access
Human Metabolome Database	111,005	5,645	48,623 *	http://www.hmdb.ca/
KEGG	3653	7298	323	http://www.genome.jp/kegg/pathway.html
Reactome	1771	11,035	2169	https://reactome.org/
WikiPathways	1421	7727	411	https://www.wikipathways.org/

* Pathways imported from the HMDB database include SMPDB and KEGG pathways.

**Table 3:** Types of queries that are supported by the web interface.

Query	Input	Tabular Output	Analysis/Visualization
Retrieve analytes for a given pathway	Pathway name(s) or pathway id(s)	Analytes that are within input pathway	
Retrieve pathway(s) for one or more analytes	Analyte name(s) or id(s)	Pathways that contain input analytes	Pathway enrichment analysis and clustering of enriched pathways
Retrieve analytes that are in the same reaction	Analyte name(s)	Analytes catalyzing or catalyzed by input analytes	Network visualization of gene-metabolite relationships
Retrieve ontologies from given metabolites	Metabolite name(s) or id(s), or ontology name	List of ontologies or metabolites that pertain to input	

## Supplementary Materials

The following are available online at http://www.mdpi.com/2218-1989/8/1/16/s1, Figure S1: Promiscuity of pathway involvement, Figure S2: Network of gene-metabolite relationships, Table S1: Information retrieved from each database, Supplementary Data: list of metabolites input into pathway overrepresentation analysis and analysis results; Supplementary Material: Step-by-step instructions on navigating the RaMP R Shiny web application.
