# Supplementary material for: RaMP: A Comprehensive Relational Database of Metabolomics Pathways for Pathway Enrichment Analysis of Genes and Metabolites
Source: Metabolites. 2018 Feb 22;8(1):16. doi: 10.3390/metabo8010016 (PMC5876005; doi:10.3390/metabo8010016)
Supplement: Supplementary file 1 [file metabolites-08-00016-s001.zip › Supplementary Information/FigS1_PathwayPromiscuity_revised.pdf]

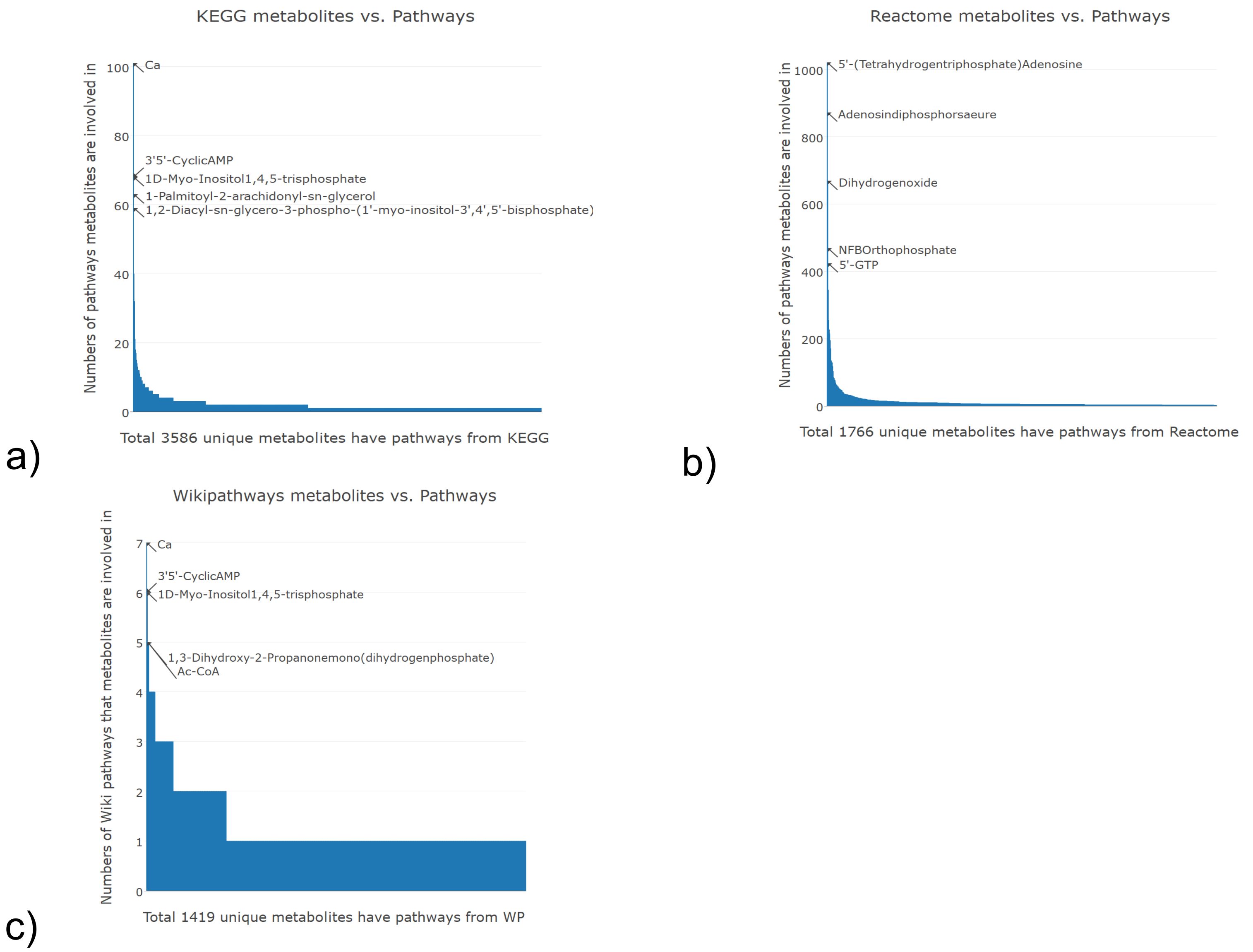

Supplementary Figure 1: Promiscuity of pathway involvement. For each metabolite, the number of pathways it belongs to is plotted for a) KEGG, b) Reactome, c) WikiPathways.
