# Supplementary material for: RaMP: A Comprehensive Relational Database of Metabolomics Pathways for Pathway Enrichment Analysis of Genes and Metabolites
Source: Metabolites. 2018 Feb 22;8(1):16. doi: 10.3390/metabo8010016 (PMC5876005; doi:10.3390/metabo8010016)
Supplement: Supplementary file 1 [file metabolites-08-00016-s001.zip › Supplementary Information/FigS2_GeneMetabNetwork.pdf]

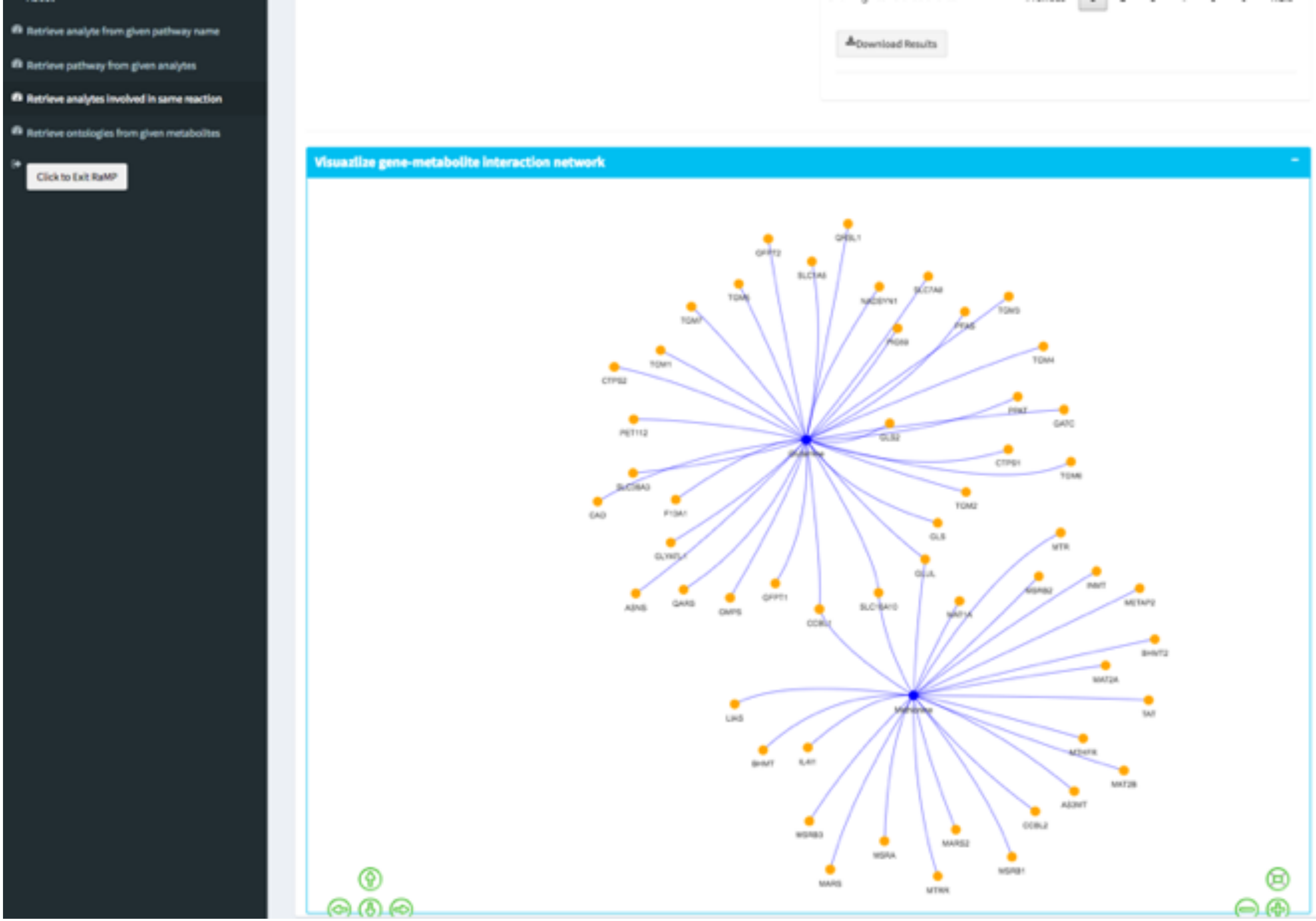

Supplementary Figure 2: Network visualization of gene-metabolite relationships, for genes that catalyze reactions involving glutamine and methionine.
