# Supplementary material for: RaMP: A Comprehensive Relational Database of Metabolomics Pathways for Pathway Enrichment Analysis of Genes and Metabolites
Source: Metabolites. 2018 Feb 22;8(1):16. doi: 10.3390/metabo8010016 (PMC5876005; doi:10.3390/metabo8010016)
Supplement: Supplementary file 1 [file metabolites-08-00016-s001.zip › Supplementary Information/SuppMaterials_WebAppInstructions_revised.docx]

***Title***: RaMP: A comprehensive Relational database of Metabolomics Pathways for pathway enrichment analysis of genes and metabolites.

***Author List and Affiliations***:

Bofei Zhang^1,3^, Senyang Hu^1,3^, Elizabeth Baskin^1,3^, Andrew Patt^1,2^, Jalal K. Siddiqui^1^, Ewy A. Mathé ^1,*^

^1^ Department of Biomedical Informatics, College of Medicine, The Ohio State University, Columbus, Ohio, United States of America

^2^ Biomedical Sciences Graduate Program, The Ohio State University, Columbus, Ohio, United States of America

^3^ These authors contributed equally

* Corresponding author; Email: ewy.mathe@osumc.edu

***Supplementary Materials*: installation instructions**

The R Shiny web application is part of the RaMP R package, which can be readily installed using the following commands in the R Console:

install.packages(“devtools”)

library(devtools)

install_github("mathelab/RAMP-DB")

Once the R package is installed, the MySQL database will need to be setup locally. Detailed instructions on doing so are available on our GitHub site https://github.com/Mathelab/RaMP-DB/.

When the R package is installed and the MySQL database is set up, the web application runs by simply typing the following in the R Console:

library(RaMP)

runRaMPapp (password=”mysqlpassword”)

Please visit <https://github.com/Mathelab/RaMP-DB/> for further details. Should you have any questions or issues, please use submit an “Issue” through the GitHub website page.

***Supplementary Materials***: **step-by-step instructions to run the RaMP web application**

RaMP pulls information from four databases, HMDB, KEGG, WikiPathways, and Reactome, including annotations for compounds, genes, pathways, and ontologies. The relationships between metabolites, pathways, and ontology are connected by their internal RaMP ID (see Methods). A user-friendly RaMP Shiny web application has been developed to facilitate access and use of the RaMP database (**Figure 1**). This Shiny application supports the following queries:

- Given a pathway name, the user can retrieve metabolites and/or genes found within the given pathway.
- Given analyte (metabolite or gene) names or source IDs, the user can retrieve a list of pathways that the analyte belongs to. This tab also supports pathway overrepresentation analysis and clustering of statistically significant pathways.
- Given a metabolite or gene, the user can retrieve a list of all genes or metabolites involved in the same reaction. This tab enables visualization of the gene-metabolite relationship network.
- Given a list of metabolite(s), the user can retrieve associated ontologies (e.g. biofluid location, etc.)

Of note, the RaMP Shiny web application supports both single and batch queries, and all tables are dynamic (allowing sorting and searching). We are continuously developing the application and will be adding more queries in the future. This document outlines the current queries available (as of February 2018) via the Shiny application.


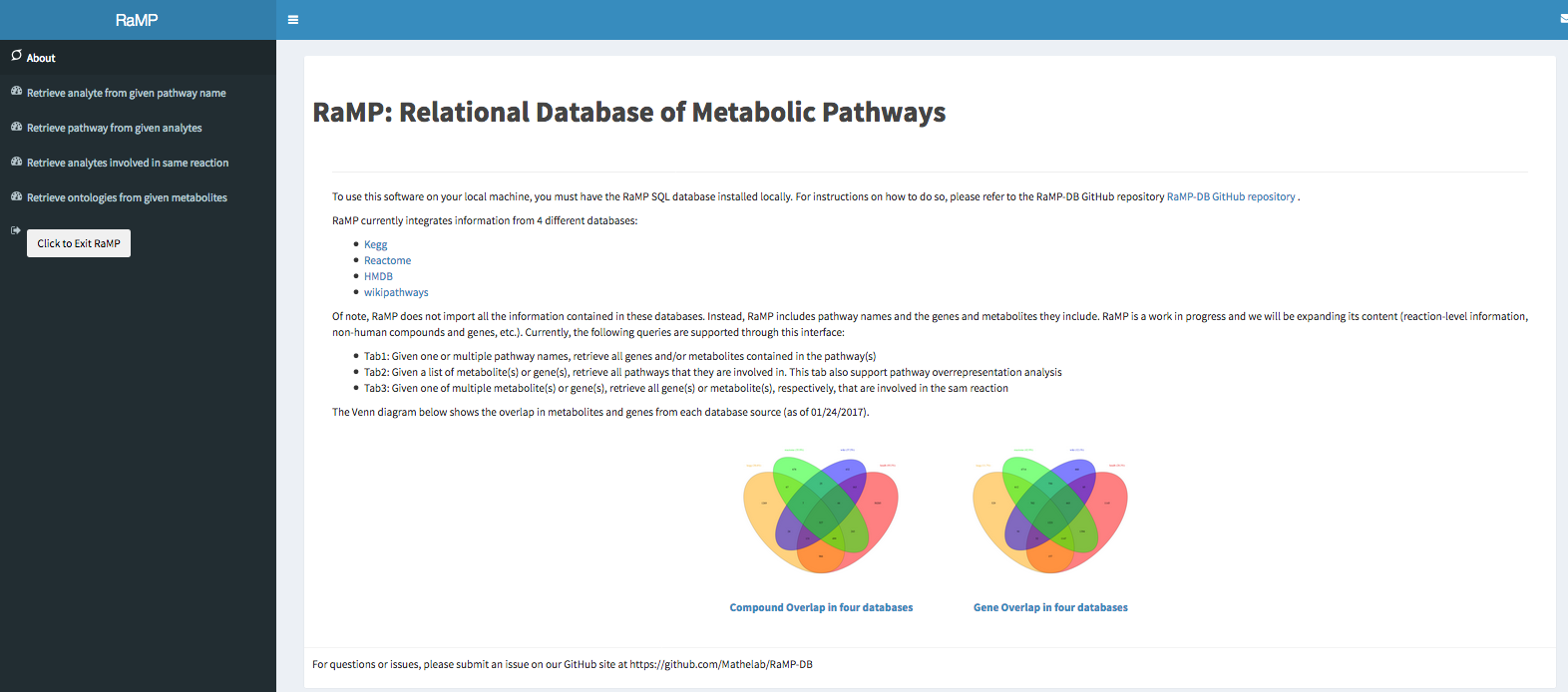


**Figure 1:** Screenshot of RaMP Shiny Web application home page.

**Query 1 tab: retrieve analytes for a given pathway**

The first tab “*Retrieve analyte from a given pathway name”* allows the user to obtain a list of metabolite and/or genes contained within a given pathway supporting both simple and batch queries (**Figure 2**). By default, the user starts in the “*Input pathway one by one”* sub-tab that supports simple queries.


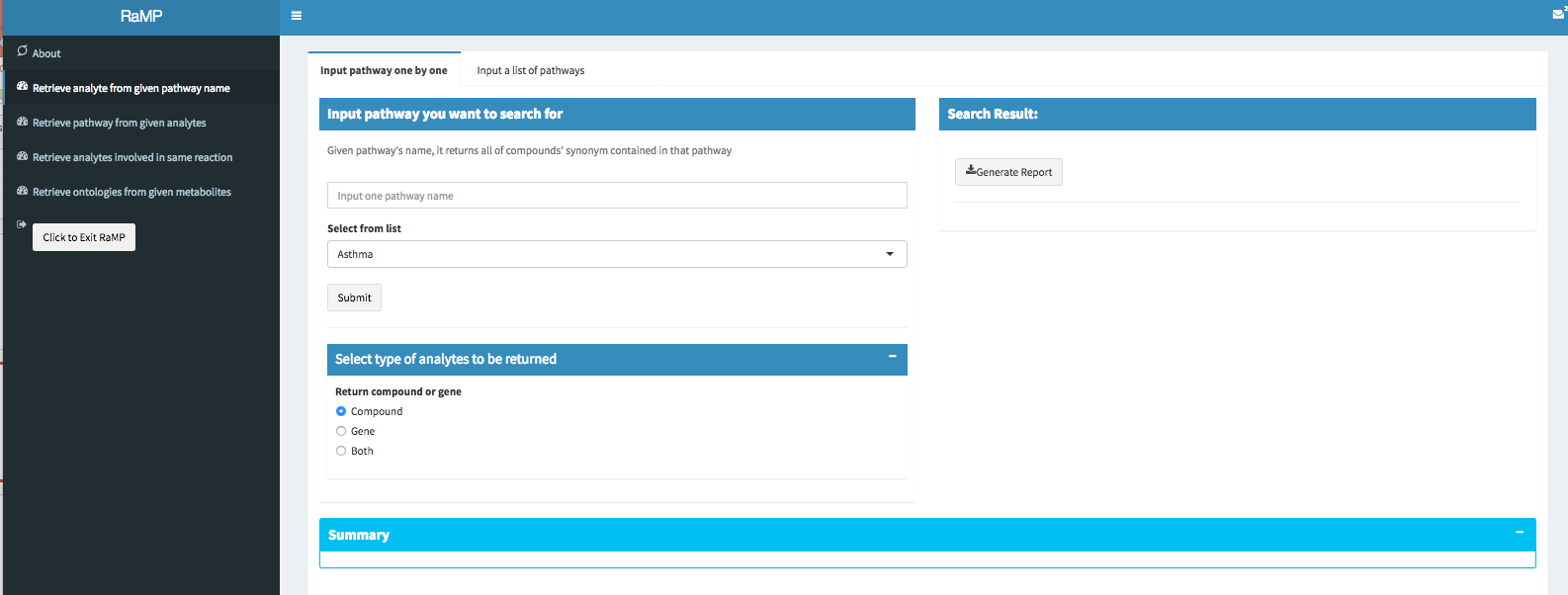


**Figure 2: RaMP tab “Retrieve analyte from given pathway name” allows users to retrieve analytes given a pathway name.**

As an example, let’s search for analytes in a pathway related to glutamine metabolism. We can type “glutamine” in the “*Input one pathway/name”* search field and the drop-down field below will list all pathways related to “glutamine” (**Figure 3**). Let’s select “*D-Glutamine and D-glutamate metabolism”* and click “*Submit”*. The user can also retrieve genes, or both genes and metabolites by checking the appropriate box on that tab.


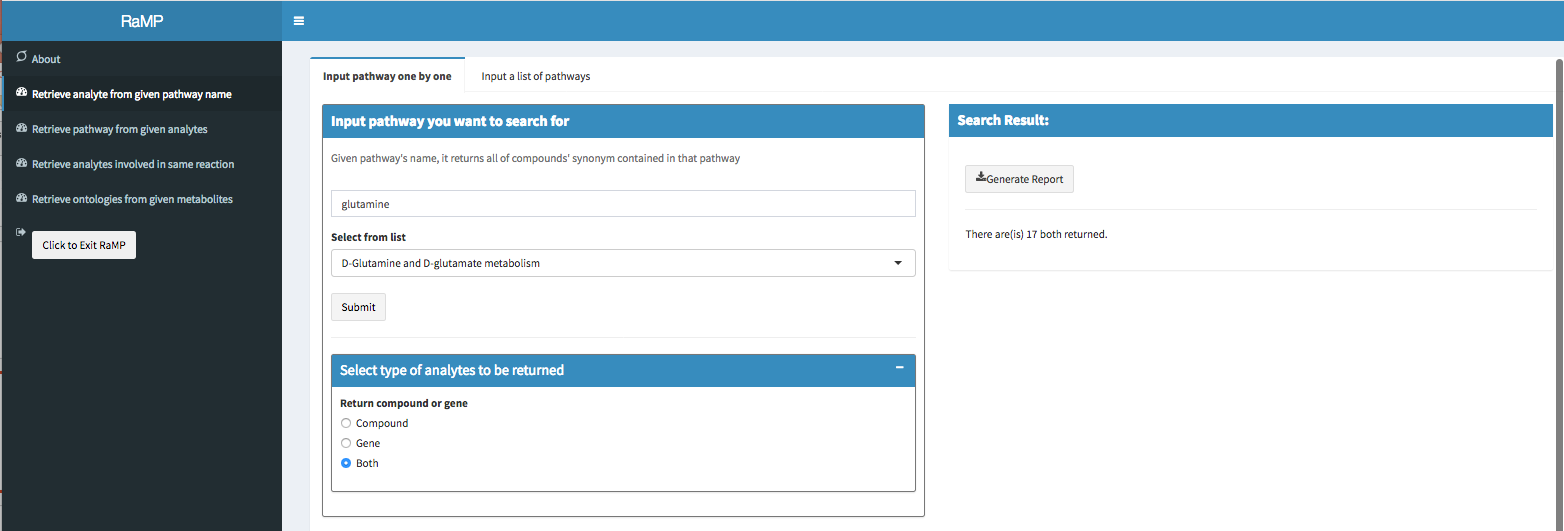


**Figure 3: Entering a pathway-related term in the tab “Retrieve analyte from given pathway name” offers a drop-down list of pathways matching that term.**

The resulting summary gives a list of compounds that are part of the *D-Glutamine and D-glutamate metabolism* pathway (**Figure 4**).


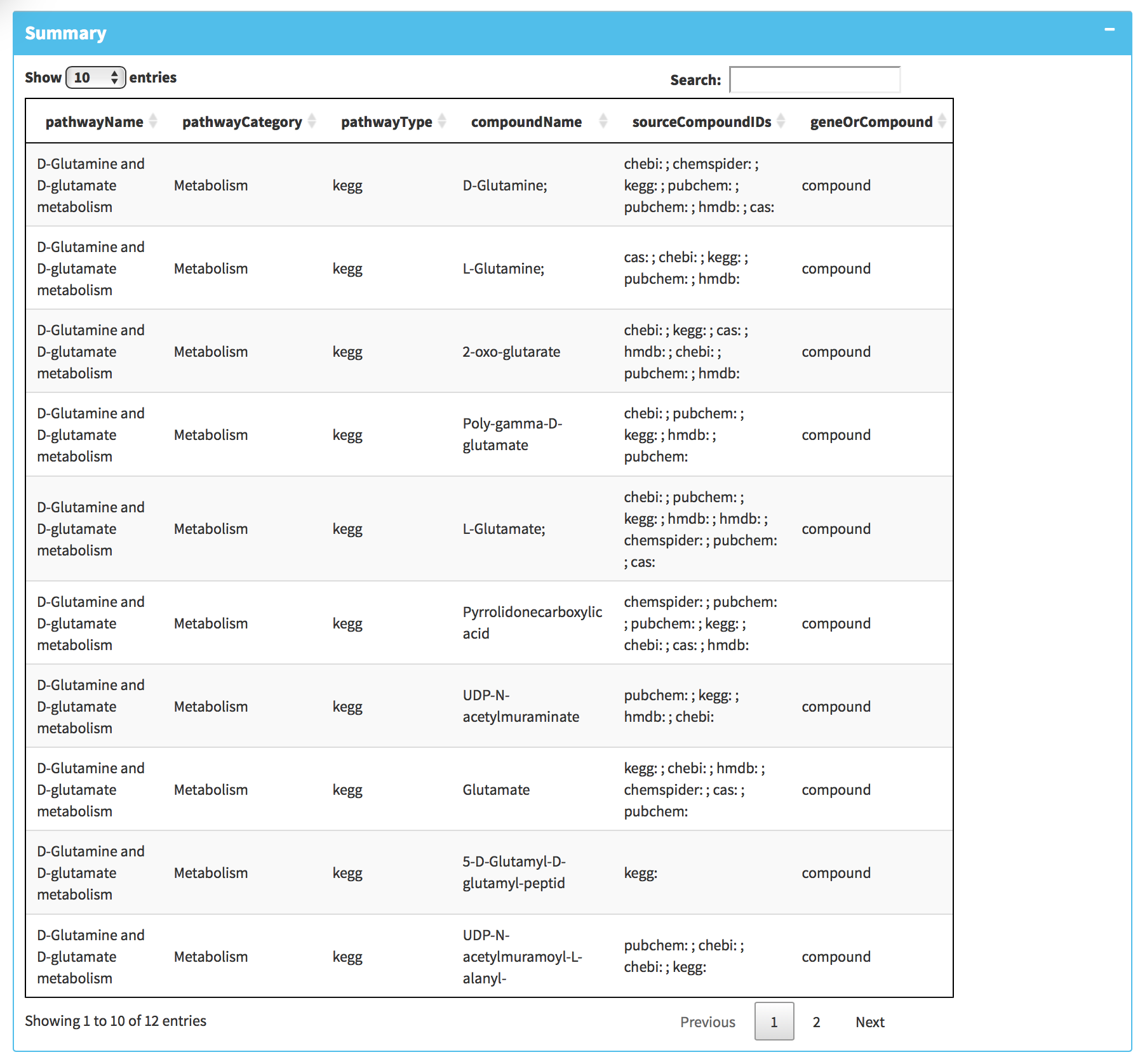


**Figure 4: RaMP Summary of compounds related to the *D-Glutamine and D-glutamate metabolism* pathway.**

The “*Input a list of pathways”* sub-tab supports batch queries, allowing the user to input a list of pathways (one per line) (**Figure 5**).


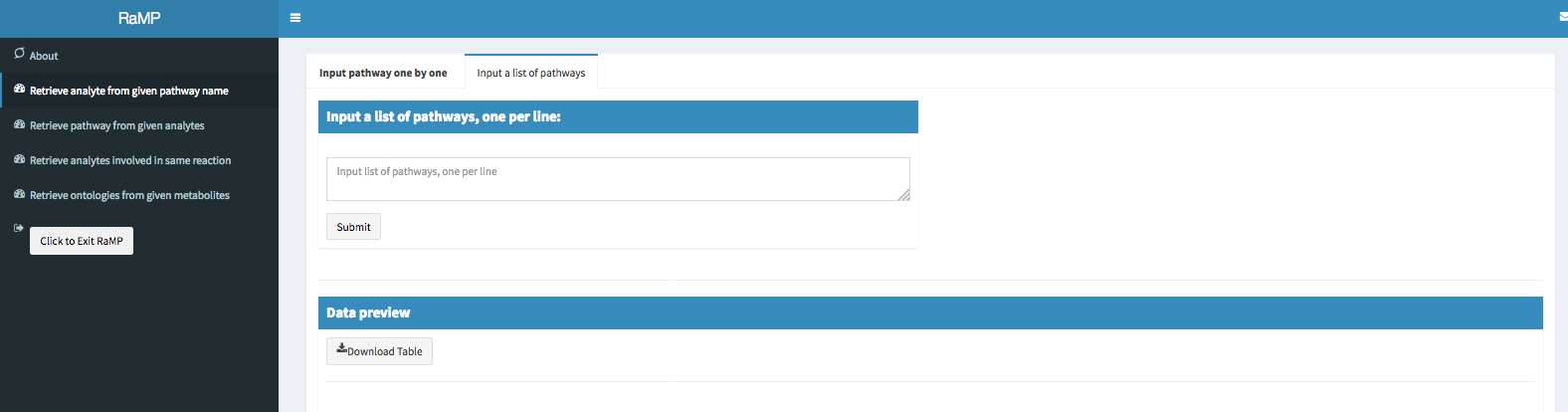


**Figure 5: The second subtab of the “Retrieve analyte from given pathway name” tab allows users to query a list of pathways and retrieve a list of genes and metabolites.**

**Query 2 tab: retrieve pathways for a given analyte(s)**

The second tab “*Retrieve pathway from given analytes”* retrieves a list of pathways given an analyte. This can be performed as a simple query or as a batch query. As a default, the “*Input analyte one by one”* sub-tab supporting simple queries is pre-selected (**Figure 6**).

**
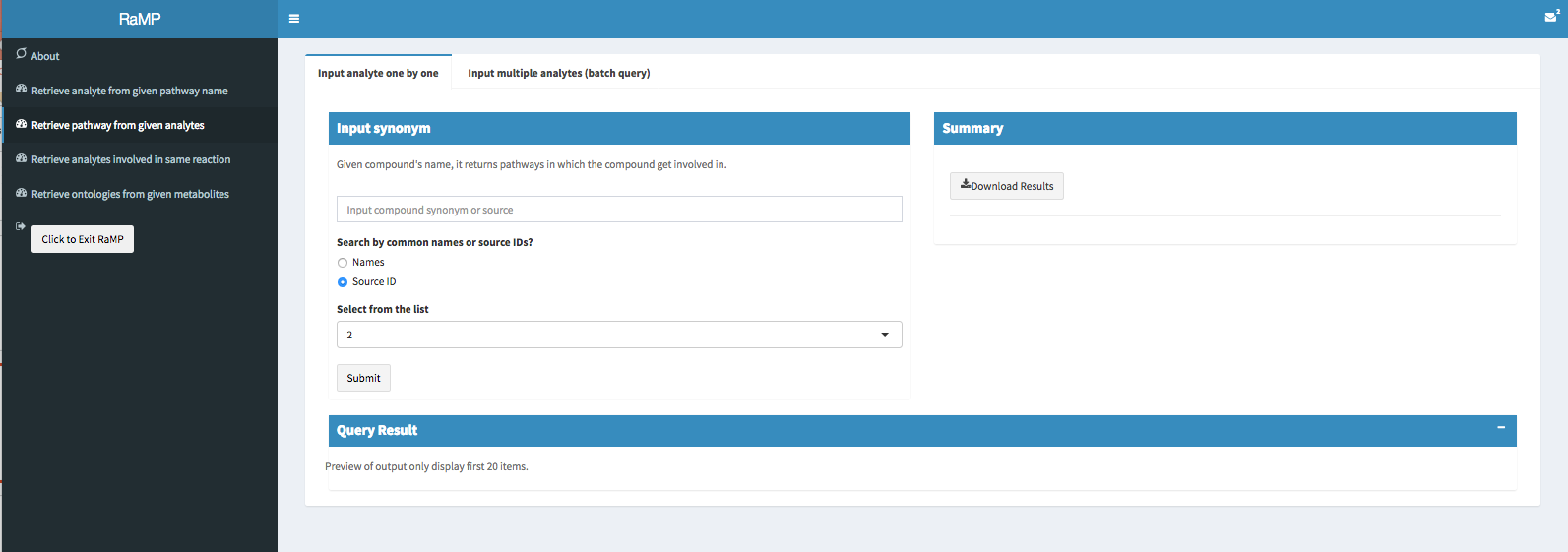
**

**Figure 6: RaMP functionality allowing user to obtain pathways given analytes.**

As an example, we enter the compound “creatine” into the “*Input compound name or source id”* search bar and select a matching compound in the drop-down list to query (**Figure 7**). In the “*Query Result”* section, we obtain a list of pathways that contain “*creatine*” as a compound (**Figure 7**). Query results can be downloaded by clicking on the “*Download Results*” button.


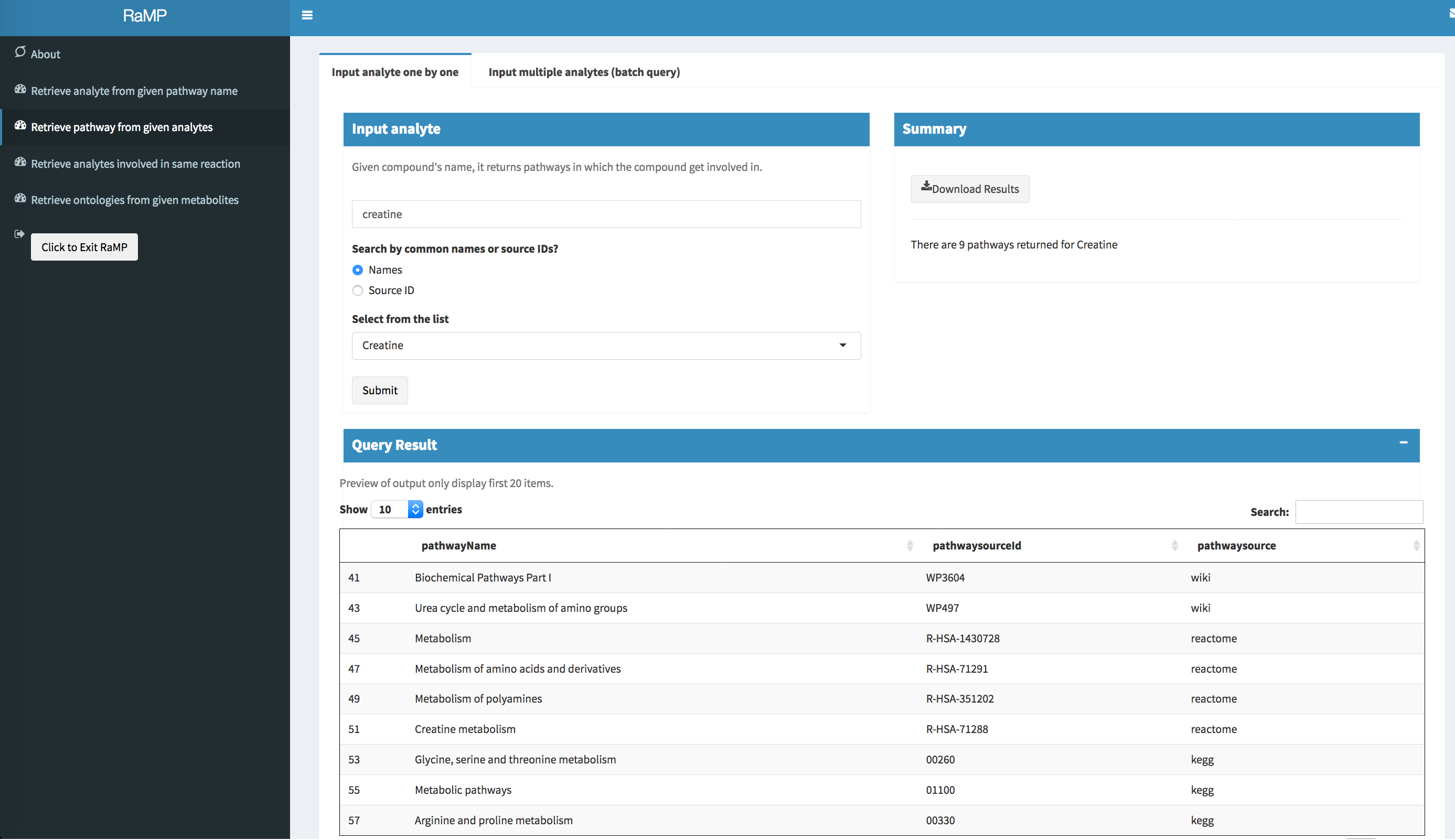


**Figure 7: Entering a compound name automatically populates a list of matching terms. In the ‘Query Result’ section, pathways containing the compound are shown.**

The “*Input Multiple analytes (batch query)”* sub-tab supports batch queries and takes a list of metabolites or genes as input to retrieve pathway information for each input analyte. This sub-tab also supports pathway enrichment analysis and functional clustering of pathways. Here we input a list of gene and metabolite names that we wish to obtain pathway information for (**Figure 8**). The “*Summary”* section shows the number of pathways retrieved per analyte (gene or metabolite) input. The resulting table that lists the pathways identified is shown under the “Query Result” section and is downloadable by clicking on the “Download Results” button.


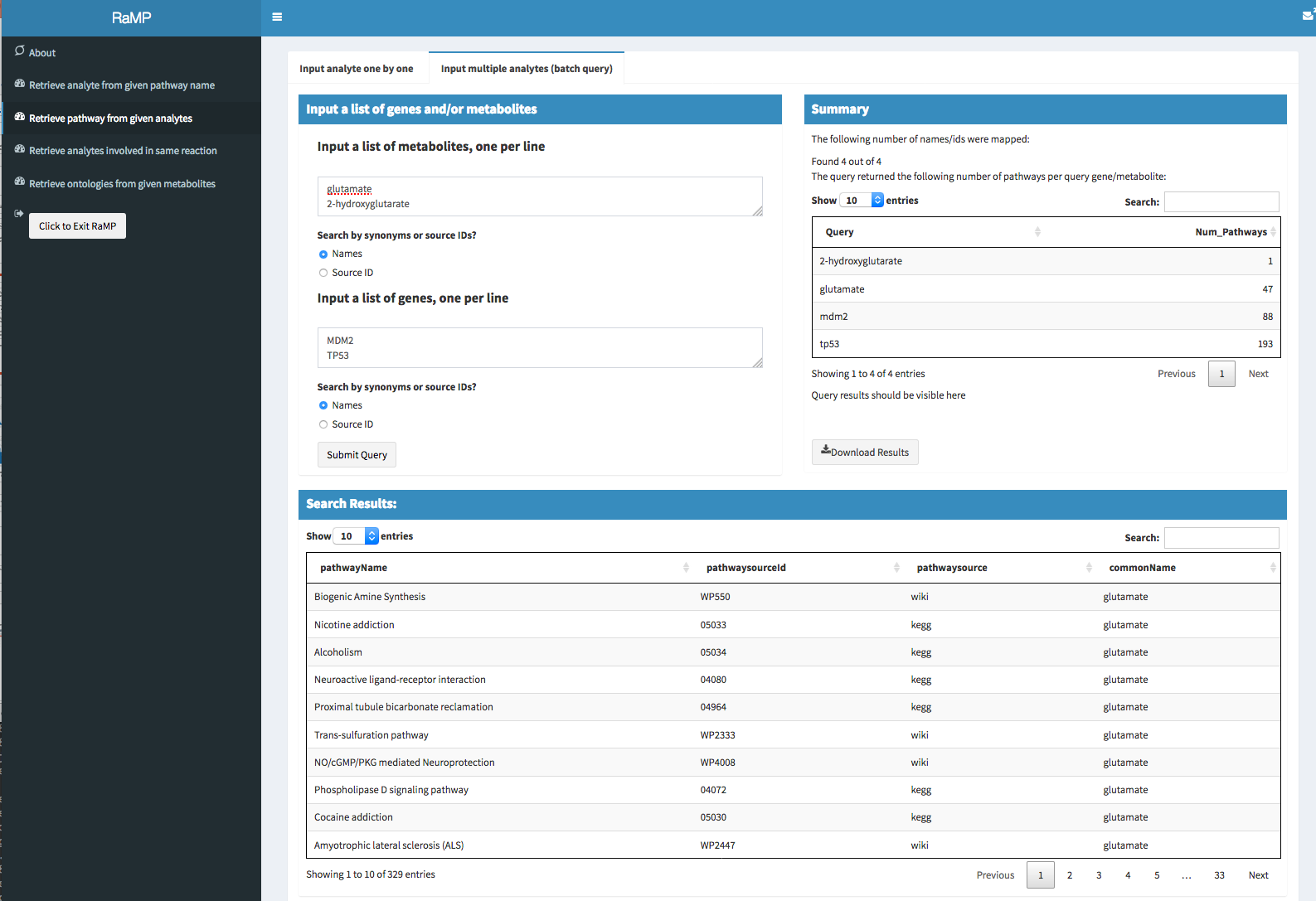


**Figure 8:** **Users can perform batch queries on a list of genes and metabolites to obtain all pathways user-input metabolites and/or genes belong in.**

RaMP also allows for pathway enrichment analysis (over-representation analysis, see Methods) on genes and/or metabolites input into the batch query. Below the “*Search Results”*, we can set parameters for the “*Pathway Enrichment Analysis”*, which we can then run (**Figure 9**).


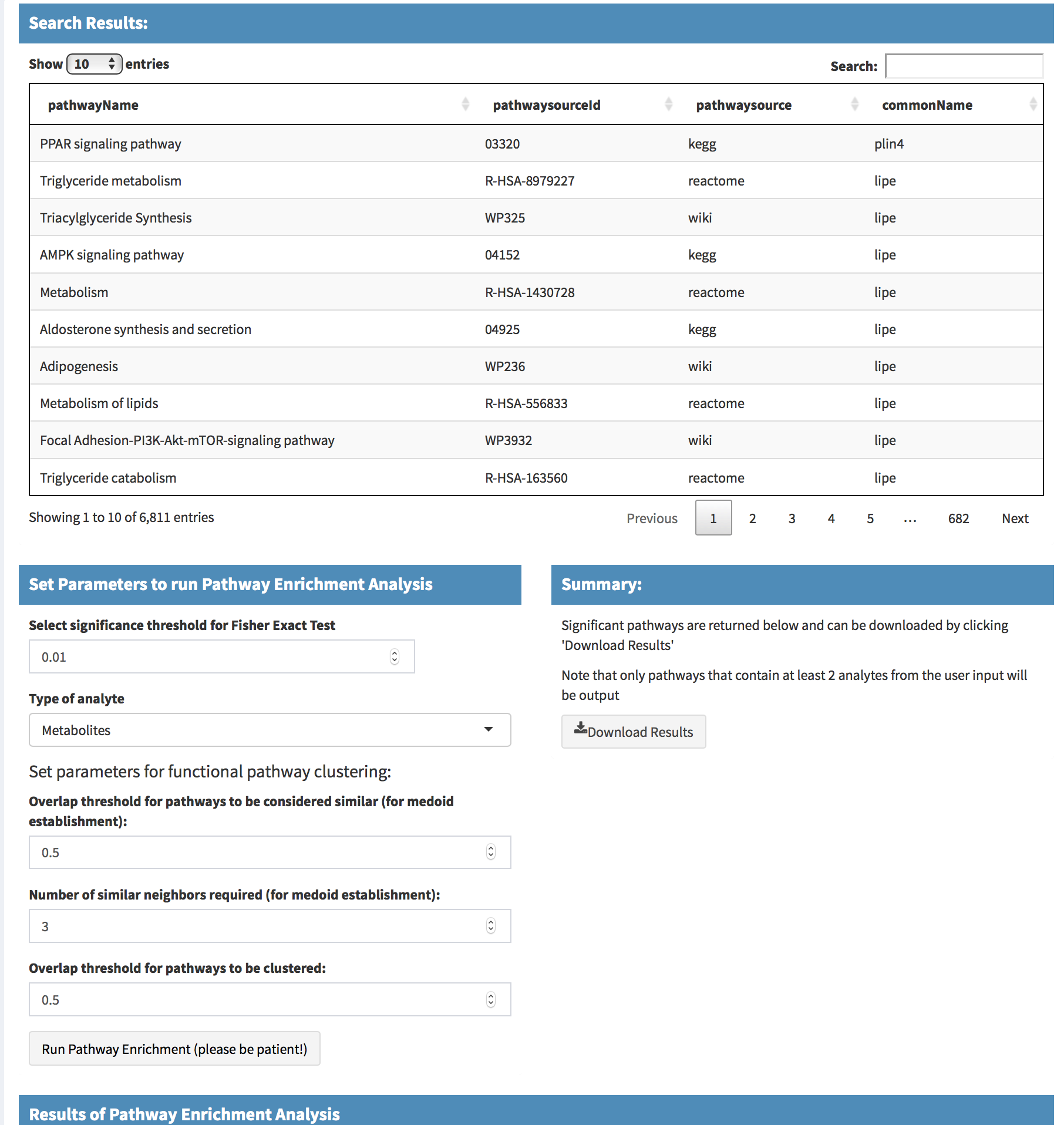


**Figure 9: RaMP allows pathway overrepresentation analysis on results of gene and/or metabolite batch query.**

The pathway analysis and functional clustering results are displayed as an interactive table that can also be downloaded (**Figure 10**).


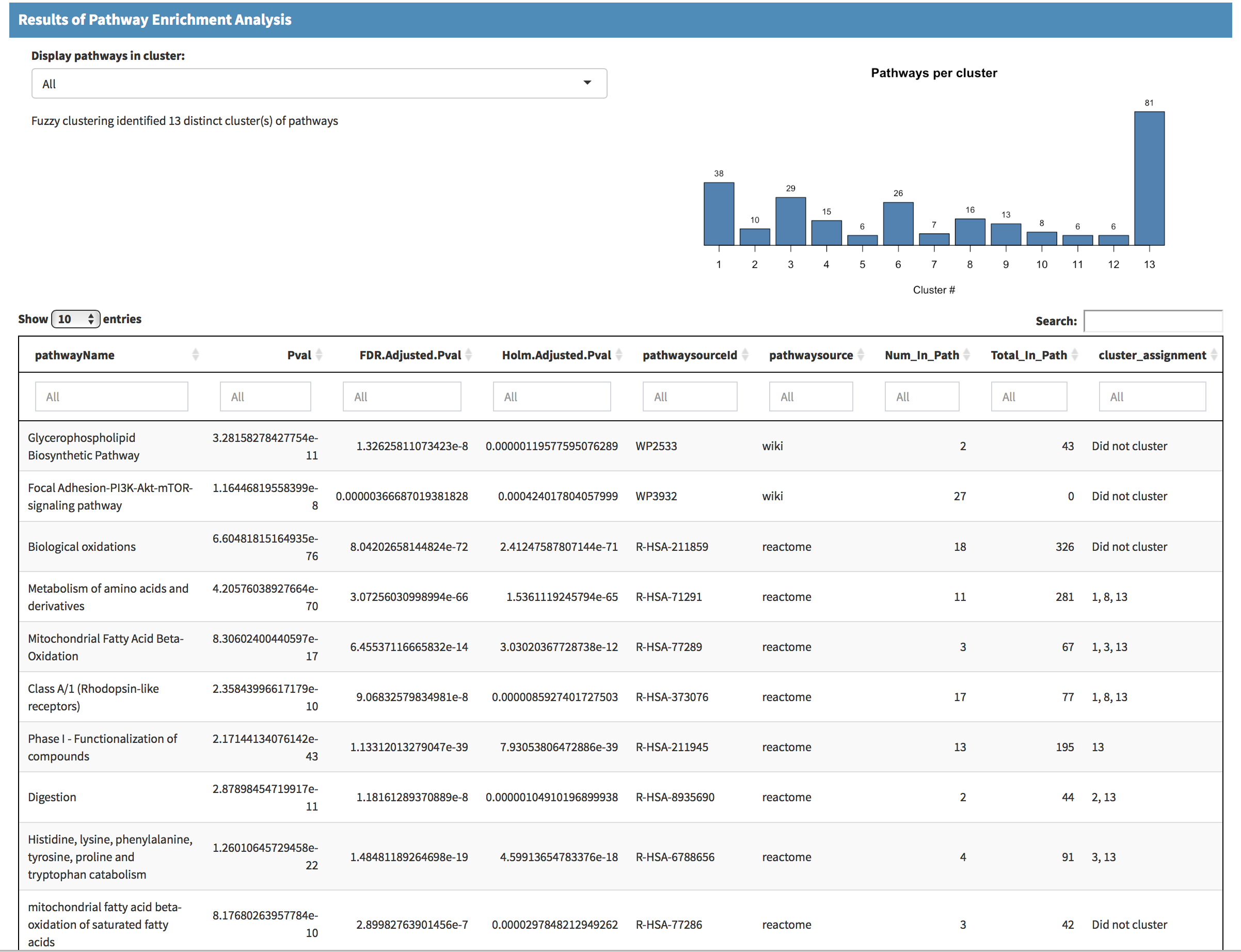


**Figure 10. Results of *Pathway Enrichment Analysis* given a list of genes.**

**Query 3 tab: retrieve gene-metabolite relationships**

The “*Retrieve metabolites or genes involved in same reaction*” tab allows users to query for genes and metabolites that are involved in the same reaction (**Figure 11**). Like previous RaMP queries, it supports both simple and batch queries.


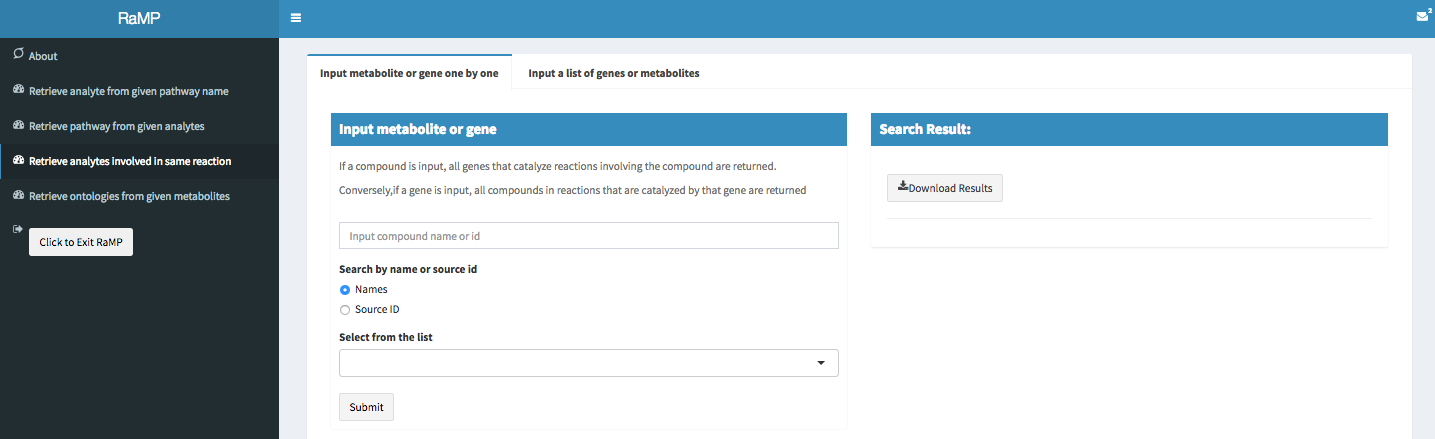


**Figure 11: This third tab retrieves analytes involved in the same reactions as input analytes.**

As an example, we are interested in retrieving compounds involved in reactions that involve glutamine. We enter “glutamine” in the “*Input metabolite or gene”* search bar and obtain a list of genes that catalyze reactions involving glutamine (**Figure 12**).


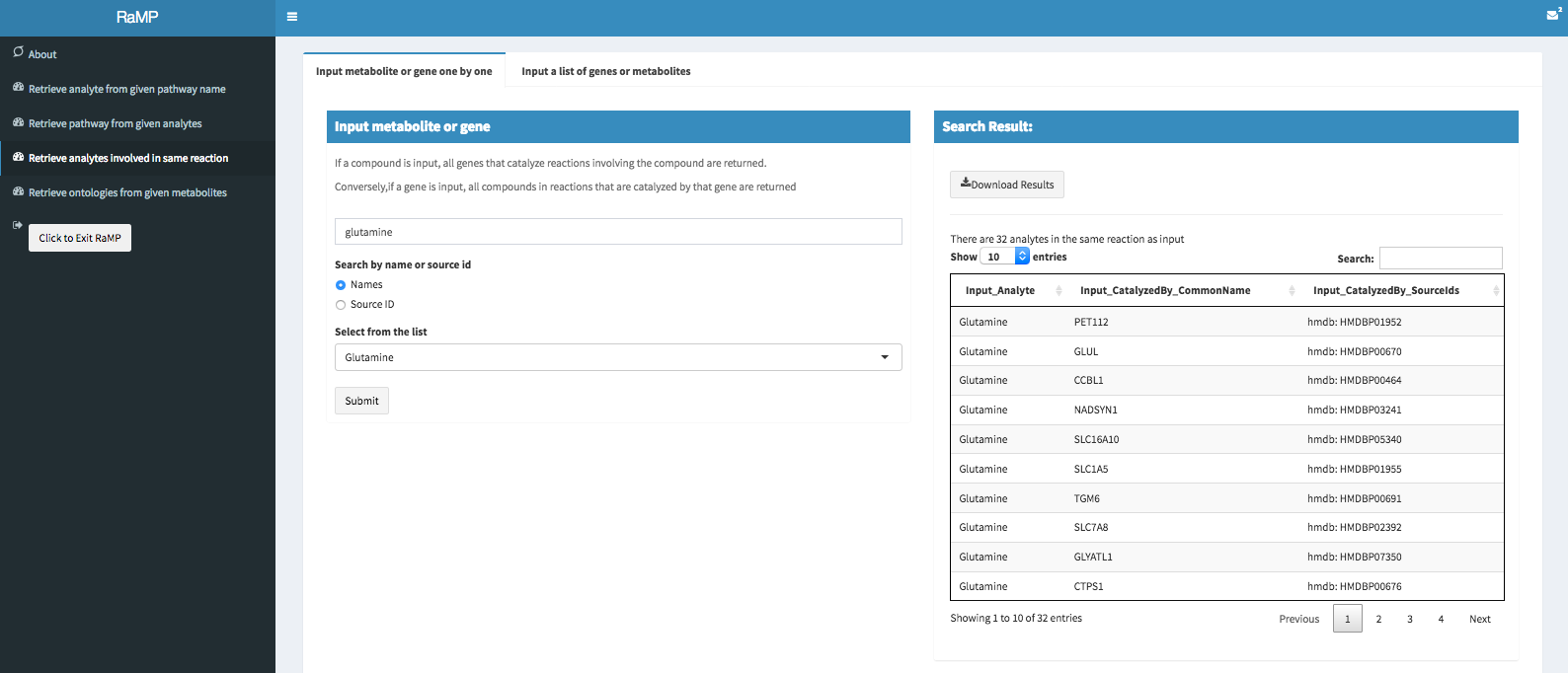


**Figure 12: Results of query for metabolites involved in reactions of glutamine.**

Users can also visualize networks of gene-metabolite relationships retrieve from queries. An example network for such relationships involving glutamine are shown in **Figure 13**.


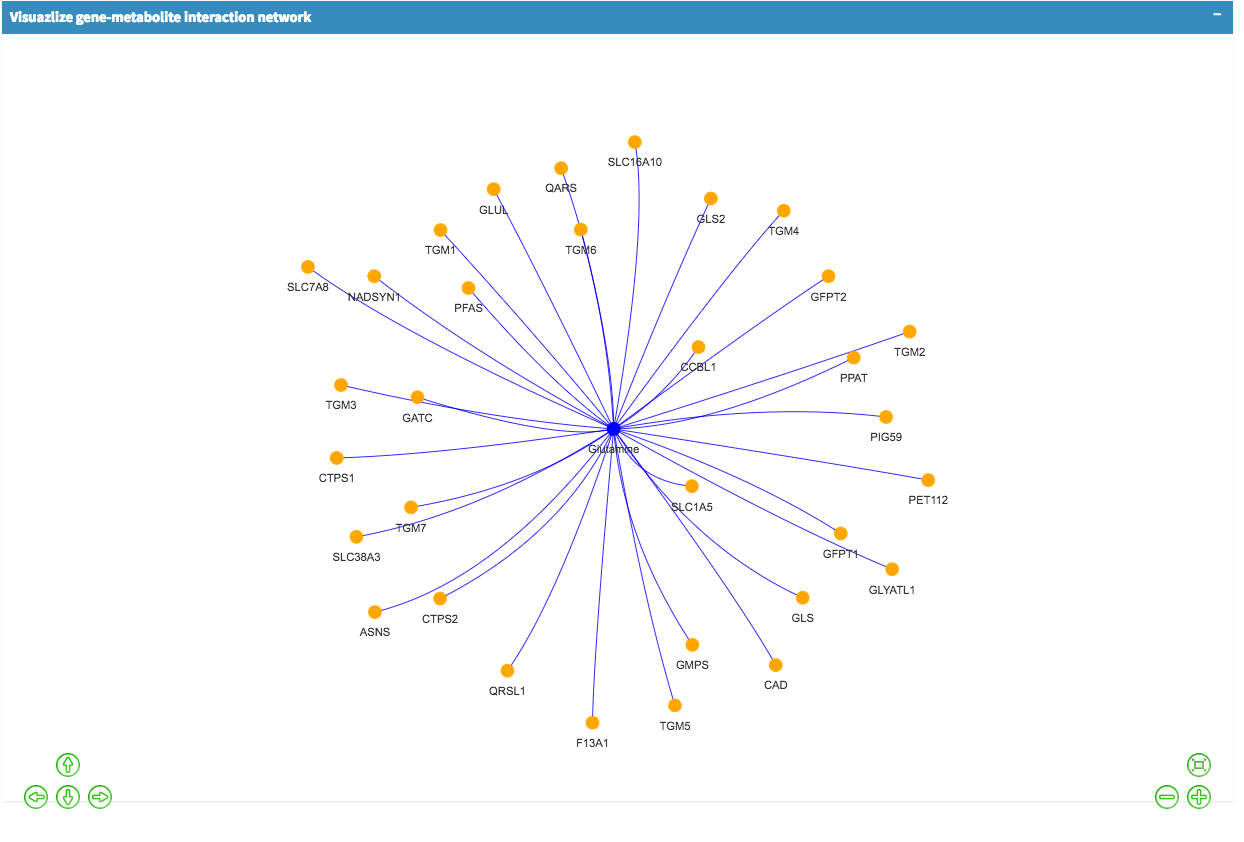


**Figure 13: Network visualization of gene-metabolite relationships.**

**Query 4 tab: retrieve ontologies**

The fourth tab “*Retrieve ontologies from given metabolites”* retrieves a list of ontologies (e.g. biofluid location, cellular location, origins, tissue location) associated with user-input metabolites (**Figure 14**). Further, this tab allows users to query an ontology, thereby retrieving all metabolites associated with that ontology. As with the other tabs, retrieval of ontologies or metabolites can be performed as a simple query or as a batch query. As a default, the “*Input analyte one by one”* sub-tab supporting simple queries is pre-selected.

**
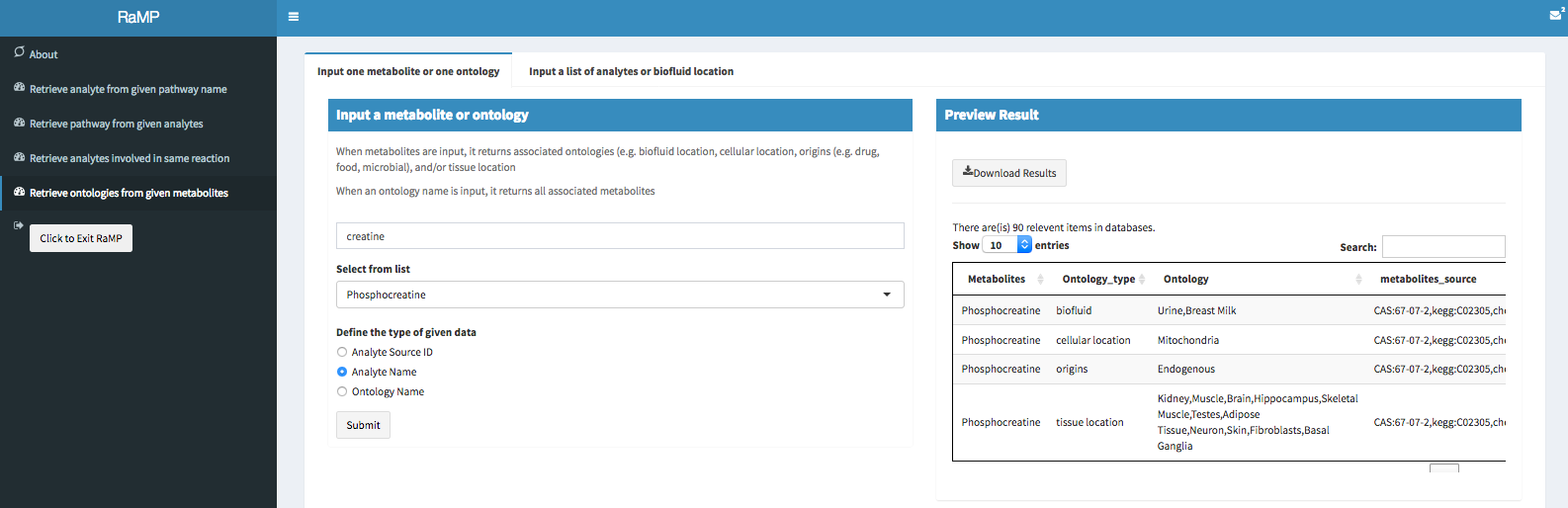
**

**Figure 14: This fourth tab retrieves ontologies related to user-input metabolites, or metabolites involved in user-input ontologies.**
