# Supplementary material for: RaMP: A Comprehensive Relational Database of Metabolomics Pathways for Pathway Enrichment Analysis of Genes and Metabolites
Source: Metabolites. 2018 Feb 22;8(1):16. doi: 10.3390/metabo8010016 (PMC5876005; doi:10.3390/metabo8010016)
Supplement: Supplementary file 1 [file metabolites-08-00016-s001.zip › Supplementary Information/TableS1_InfoRetrievedFromSourceDBs.pdf]

Table S1: List of IDs and other information retrieved from each database.

Database	Compound Information	Gene Information	Pathway Information
KEGG	• ID (prefix “C”)<br>• Synonyms<br>• Other IDs (ChEBI, CAS)	• ID (prefix “hsa”)<br>• Other IDs (UniProt, KEGG, Ensembl, HUGO name)	• ID (prefix ”map” and “hsa”)<br>• Name
HMDB	• ID (prefix “HMDB”)<br>• Synonyms<br>• Other IDs (ChEBI,DrugBank, ChemSpider, KEGG,BioCyc, PubChem compound, CAS)	• ID (UniProt)<br>• Other IDs (HUGO name)	• ID (SMPDB id)<br>• Name<br>• Ontologies (biofluid type, cellular location, origins, tissue location)
Reactome	• ID (ChEBI ID)	• ID (UniProt)	• ID (prefix “R-HSA-”)<br>• Name
WikiPathways	• ID<br>• Other IDs (HMDB, KEGG, PubChem compound, ChEBI, CAS, ChemSpider)	• ID<br>• Other IDs (UniProt, Entrez, Ensembl)	• ID (prefix “WP”)<br>• Name<br>• Category (e.g. Metabolism)
